# Ohio College of Dental Surgery

**Published:** 1865-07

**Authors:** 


### OHIO COLLEGE OF DENTAL SURGERY.

The Twentieth Annual Announcement of this Institution was recently issued.

By it, it will be seen there has been an entire reorganization of the Faculty, by which we trust none of the former efficiency will be lost. The facility for giving instruction in the different departments is not excelled.

